# Anticancer Activities of Six Selected Natural Compounds of Some Cameroonian Medicinal Plants

**DOI:** 10.1371/journal.pone.0021762

**Published:** 2011-08-19

**Authors:** Victor Kuete, Hippolyte K. Wabo, Kenneth O. Eyong, Michel T. Feussi, Benjamin Wiench, Benjamin Krusche, Pierre Tane, Gabriel N. Folefoc, Thomas Efferth

**Affiliations:** 1 Department of Biochemistry, Faculty of Science, University of Dschang, Dschang, Cameroon; 2 Department of Pharmaceutical Biology, Institute of Pharmacy and Biochemistry, University of Mainz, Mainz, Germany; 3 Department of Chemistry, Faculty of Science, University of Dschang, Dschang, Cameroon; 4 Department of Organic Chemistry, Faculty of Science, University of Yaoundé I, Yaoundé, Cameroon; Biological Research Center of the Hungarian Academy of Sciences, Hungary

## Abstract

**Background:**

Natural products are well recognized as sources of drugs in several human ailments. In the present work, we carried out a preliminary screening of six natural compounds, xanthone V_1_ (**1**); 2-acetylfuro-1,4-naphthoquinone (**2**); physcion (**3**); bisvismiaquinone (**4**); vismiaquinone (**5**); 1,8-dihydroxy-3-geranyloxy-6-methylanthraquinone (**6**) against MiaPaCa-2 pancreatic and CCRF-CEM leukemia cells and their multidrug-resistant subline, CEM/ADR5000. Compounds **1** and **2** were then tested in several other cancer cells and their possible mode of action were investigated.

**Methodology/Findings:**

The tested compounds were previously isolated from the Cameroonian medicinal plants *Vismia laurentii* (**1**, **3**, **4**, **5** and **6**) and *Newbouldia laevis* (**2**). The preliminary cytotoxicity results allowed the selection of xanthone V_1_ and 2-acetylfuro-1,4-naphthoquinone, which were then tested on a panel of cancer cell lines. The study was also extended to the analysis of cell cycle distribution, apoptosis induction, caspase 3/7 activation and the anti-angiogenic properties of xanthone V_1_ and 2-acetylfuro-1,4-naphthoquinone. IC_50_ values around or below 4 µg/ml were obtained on 64.29% and 78.57% of the tested cancer cell lines for xanthone V_1_ and 2-acetylfuro-1,4-naphthoquinone, respectively. The most sensitive cell lines (IC_50_<1 µg/ml) were breast MCF-7 (to xanthone V_1_), cervix HeLa and Caski (to xanthone V_1_ and 2-acetylfuro-1,4-naphthoquinone), leukemia PF-382 and melanoma colo-38 (to 2-acetylfuro-1,4-naphthoquinone). The two compounds showed respectively, 65.8% and 59.6% inhibition of the growth of blood capillaries on the chorioallantoic membrane of quail eggs in the anti-angiogenic assay. Upon treatment with two fold IC_50_ and after 72 h, the two compounds induced cell cycle arrest in S-phase, and also significant apoptosis in CCRF-CEM leukemia cells. Caspase 3/7 was activated by xanthone V_1_.

**Conclusions/Significance:**

The overall results of the present study provided evidence for the cytotoxicity of compounds xanthone V_1_ and 2-acetylfuro-1,4-naphthoquinone, and bring supportive data for future investigations that will lead to their use in cancer therapy.

## Introduction

Natural products are well recognized as sources for drugs in several human ailments including cancers. Examples of natural pharmaceuticals from plants include vincristine, irinotecan, etoposide and paclitaxel [1]. Despite the discovery of many drugs of natural origin, the search for new anticancer agents is still necessary, in order to increase the range available and to find less toxic and more effective drugs. It has been recommended that samples with pharmacological usage should be taken into account when selecting plants to treat cancer, as several ailments reflect disease states bearing relevance to cancer or cancer-like symptoms [2], [3]. Therefore, we designed the present work to investigate the cytotoxicity of six natural compounds available in our research group, with previously demonstrated pharmacological activities. Compound **1** has been isolated from the roots of *Cratoxylum formosum* [4], the leaves of *Symphonia globulifera* [5], and the seeds of *Vismia laurentii* [6]. The only reported natural source of compound **2** is *Newbouldia laevis* in which it can be isolated from the roots [7]. Compound **3** is a key active ingredient of the ethanol extract from roots of Chinese rhubarb (*Rheum officinale* Baill.) that has been commercialised in China for controlling powdery mildews [8]. Compound **3** was purified from several plants including *Rumex japonicus* [9], *Radix Boehmeriae* [10], *Discocleidion rufescens* [11], *Senna septemtrionalis* [12], etc. Compounds **4** and **5** are mostly found in plants of the genus *Vismia* [13], [14], whilst compound **6** was reported in *Vimia laurentii* [13] and *Psorospermum* species [15]. Compounds **2**; **4**; **5** and **6** previously showed antimicrobial activities against a panel of bacteria and fungi [13], [16]; compound **3** exhibited antibacterial activities against *Chlorella fusa* and *Bacillus megaterium*, respectively [6] and compound **1** showed antileishmanial [5] and cytotoxic activities against HeLa, HT-29 and KB cell lines [4]. Compound **1** also exhibited significant antibacterial activities on *Pseudomonas aeruginosa*, *Bacillus cereus, Staphylococcus aureus*, *Streptococcus faecalis* and *Salmonella typhi* with minimal inhibitory concentration below 10 µg/ml [4]. In the present work, we examined at first, the cytotoxicity of compounds **1**–**6** against MiaPaCa-2, CCRF-CEM, and CEM/ADR5000 cell lines, then we selected compounds **1** and **2** which were tested on a panel of cancer cells. Their possible modes of action were also investigated and reported herein.

## Results and Discussion

**Figure 1:**
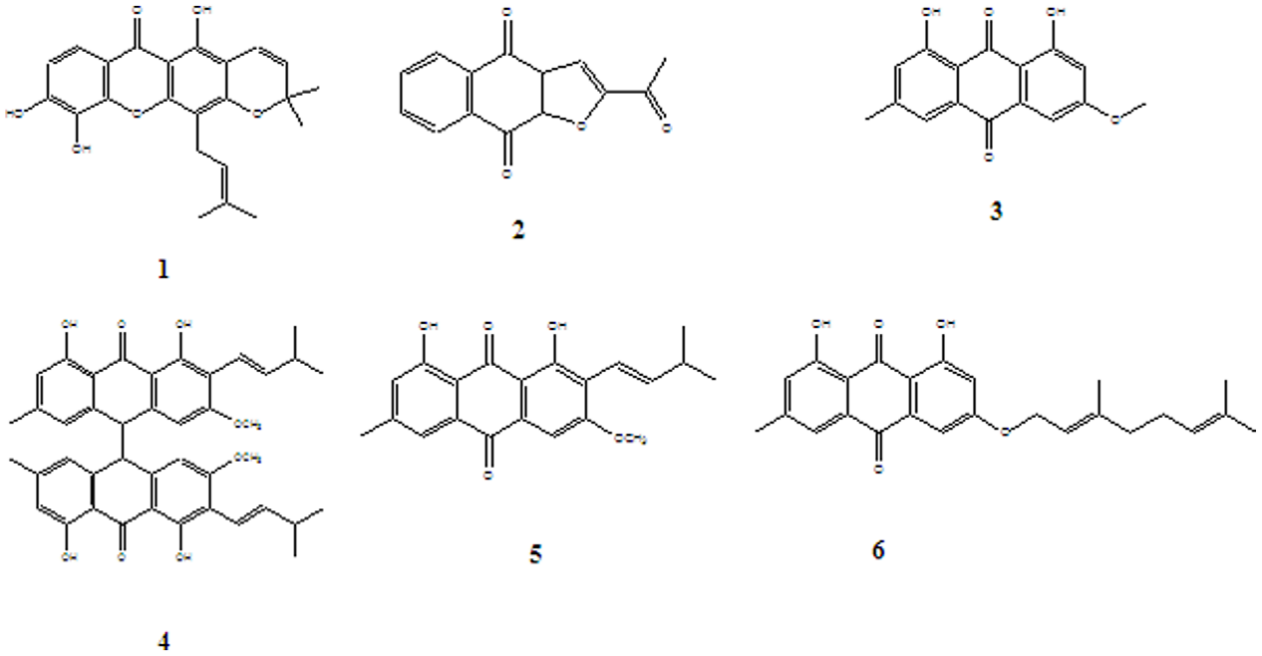
Chemical structures of the studied compounds. **1:** xanthone V_1_, **2:** 2-acetylfuro-1,4-naphthoquinone; **3:** physcion; **4:** bisvismiaquinone; **5:** vismiaquinone; **6:** 1,8-dihydroxy-3-geranyloxy-6-methylanthraquinone.

**Figure 2:**
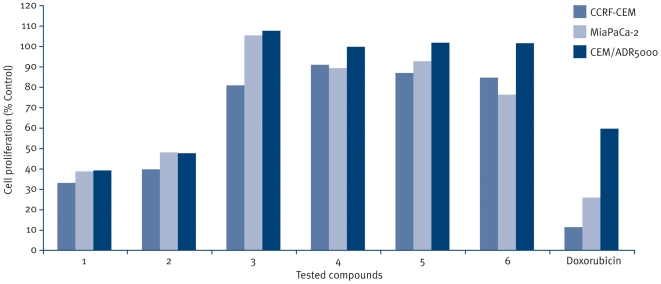
Growth percentage (%) of compounds and doxorubicin tested at 20 µg/ml on CCRF-CEM, CEM/ADR5000 and MiaPaCa-2 cell lines. **1:** xanthone V_1_, **2:** 2-acetylfuro-1,4-naphthoquinone; **3:** physcion; **4:** bisvismiaquinone; **5:** vismiaquinone; **6:** 1,8-dihydroxy-3-geranyloxy-6-methylanthraquinone. Data with different superscript letters are significantly different (P<0.05).

**Figure 3:**
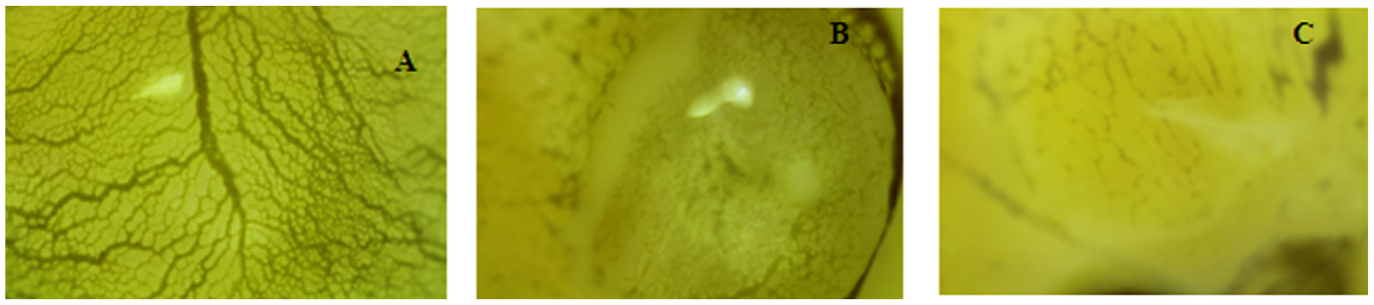
Effects of compound 1 and 2 (at 20 µg/ml) on the growth of blood capillaries on the chorioallantoic membrane of quail eggs. (A) DMSO (Control): growth of blood capillaries on the CAM – no antiangiogenic effect; (B): compound **1** (65.8% inhibition); (C): compound **2** (59.6% inhibition). Quantitative analysis was performed using a software routine which was written in the Image J-macro language, and the total small vessels number was then determined by the system as 44644 (control), 15273 (compound **1**) and 18050 (compound **2**). The inhibition percentage was then calculated as previously described [27].

**Figure 4:**
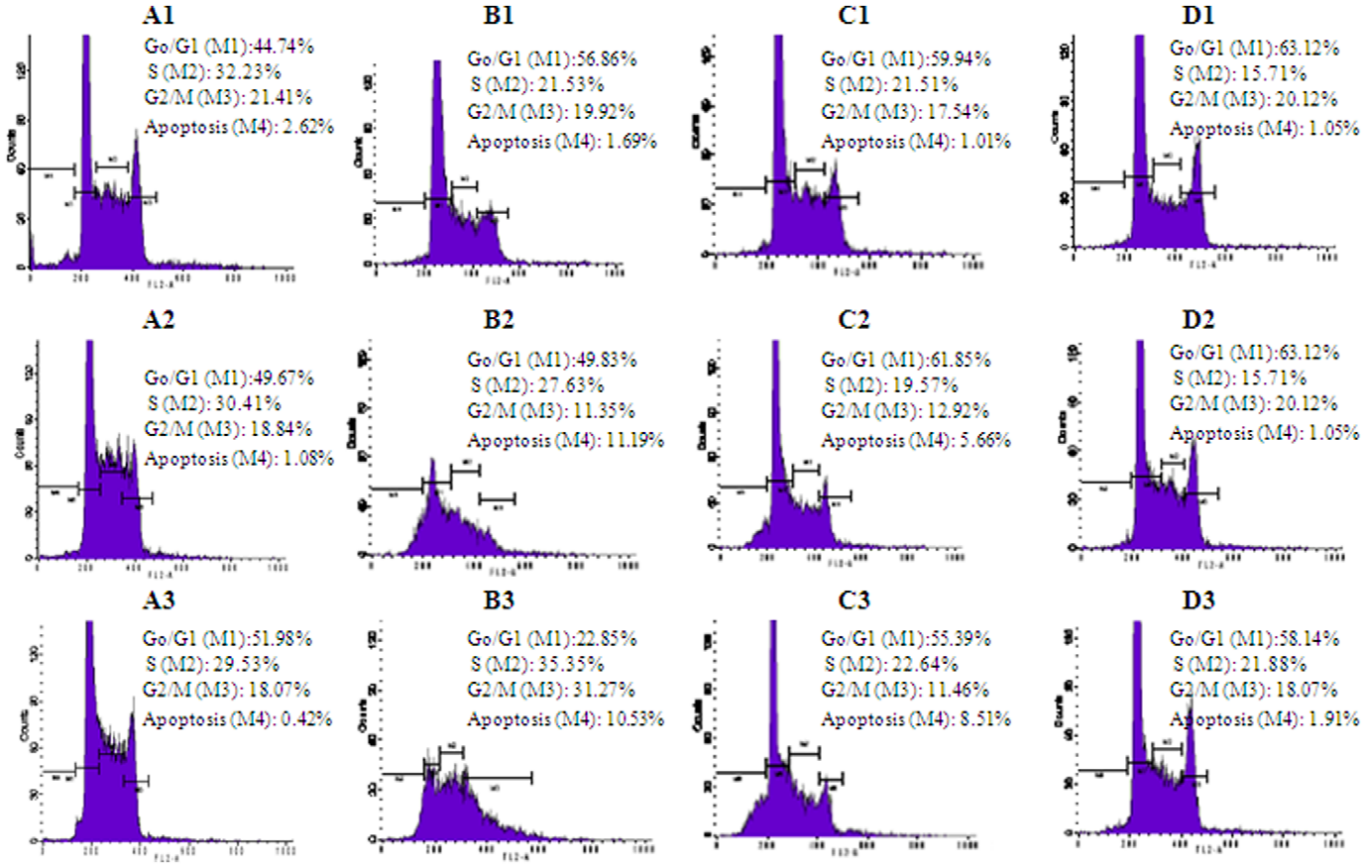
Cell cycle distribution with Leukemia CCRF-CEM treated with compounds 1. **A1**: control after 24 h; **A2**: control after 48 h; **A3**: control after 72 h; **B1**: treated with 2×IC_50_ after 24 h; **B2**: treated with 2×IC_50_ after 48 h; **B3**: treated with 2×IC_50_ after 72 h; **C1**: treated with 2×IC_50_ after 24 h; **C2**: treated with 2×IC_50_ after 48 h; **C3**: treated with 2×IC_50_ after 72 h; **D1**: treated with 2×IC_50_ after 24 h; **D2**: treated with 2×IC_50_ after 48 h; **D3**: treated with 2×IC_50_ after 72 h.

**Figure 5:**
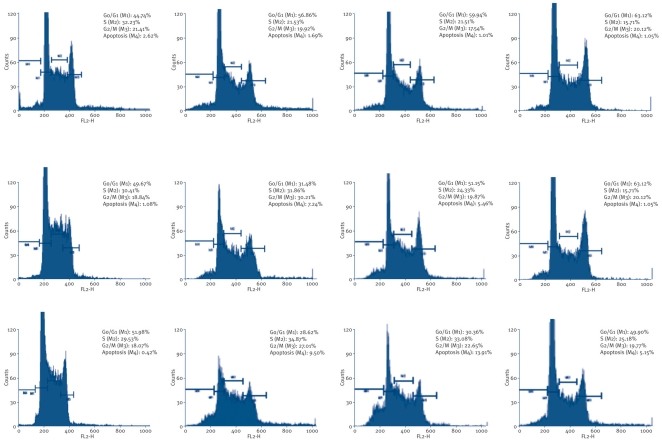
Cell cycle distribution with Leukemia CCRF-CEM treated with compounds 2. **A1**: control after 24 h; **A2**: control after 48 h; **A3**: control after 72 h; **B1**: treated with 2×IC_50_ after 24 h; **B2**: treated with 2×IC_50_ after 48 h; **B3**: treated with 2×IC_50_ after 72 h; **C1**: treated with 2×IC_50_ after 24 h; **C2**: treated with 2×IC_50_ after 48 h; **C3**: treated with 2×IC_50_ after 72 h; **D1**: treated with 2×IC_50_ after 24 h; **D2**: treated with 2×IC_50_ after 48 h; **D3**: treated with 2×IC_50_ after 72 h.

**Figure 6:**
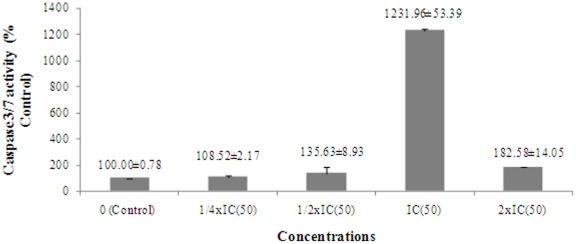
Enzymatic activity of caspase 3/7 after 6 h treatment of CCRF-CEM cells with compounds 1. The activity of caspase 3/7 is expressed as percentage % relative to untreated cells. Compound **2** did not show any induction of caspase 3/7 activity (data not shown). Values are mean ± SD of three duplicated experiments.

The six naturally occuring compounds tested included one xanthone named xanthone V_1_ (**1**) and five quinones amongst which were three anthraquinones known as physcion (**3**); vismiaquinone (**5**) and 1,8-dihydroxy-3-geranyloxy-6-methylanthraquinone (**6**), one naphthoquinone known as 2-acetylfuro-1,4-naphthoquinone (**2**) and one binaphthoquinone named bisvismiaquinone (**4**) (Fig. 1). The four studied naphtoquinones have compound **3** as the basic moiety. The preliminary cytotoxicity of the six studied compounds on CCRF-CEM, CEM/ADR5000 and MiaPaca-2 is summarized in Fig. 2. Only xanthone V_1_ and 2-acetylfuro-1,4-naphthoquinone as well as doxorubicin were able to reduce the proliferation of the three cell lines by up to 50%, when tested at 20 µg/ml. Physcion was previously found to have no cytotoxic activity on some cancer cell lines such as K562, HeLa, Calu-1, Wish and Raji [17] and was not significantly active as observed in the present work. However, the low activity of 8-dihydroxy-3-geranyloxy-6-methylanthraquinone (**6**), as well as bisvismiaquinone (**4**) and vismiaquinone (**5**) also bearing physcion (**3**) moiety, clearly highlights the low cytotoxicity of the studied anthraquinones. It can be deduced that the best cytotoxic activity of the studied compounds were obtained with the tested xanthone (xanthone V_1_) and naphthoquinone (2-acetylfuro-1,4-naphthoquinone). Xanthone V_1_ and 2-acetylfuro-1,4-naphthoquinone were therefore selected and tested on several cancer cells. The IC_50_ values obtained are reported in Table 1 and values below 20 µg/ml were recorded on 12 of the 14 (85.71%) tested cancer cell lines for xanthone V_1_ and 14/14 (100%) for 2-acetylfuro-1,4-naphthoquinone. Considering the cut-off points of 4 µg/ml [18] or 10 µM [19] for good cytotoxic compounds, values around or below this set point were obtained by xanthone V_1_ on 9/14 (64.29%) tested cancer cell lines and 11/14 (78.57%) for 2-acetylfuro-1,4-naphthoquinone. The most sensitive cell lines (with IC_50_ values below 1 µg/ml) were breast MCF-7 (to xanthone V_1_) and cervix HeLa and Caski (to xanthone V_1_ and 2-acetylfuro-1,4-naphthoquinone), leukemia PF-382 and melanoma colo-38 (to 2-acetylfuro-1,4-naphthoquinone). The liver is the main organ involved in drug metabolism. Therefore AML12 hepatocyte were choosen in the present work to evaluate the cytotoxicity of the compounds on non cancer cells. Interrestingly, the two compounds were generally less toxic on AML12 cells, the IC_50_ being above 20 µg/ml. In addition, the two compounds showed respectively, 65.8% and 59.6% inhibition of the growth of blood capillaries on the chorioallantoic membrane of quail eggs in the anti-angiogenic assay (Fig. 3), suggesting that negative effect on tumor promotion *in vivo* could be expected. To the best of our knowledge, the anticancer activity of 2-acetylfuro-1,4-naphthoquinone is being reported herein for the first time meanwhile the cytotoxicity of xanthone V_1_ was reported on HeLa, HT-29 and KB cell lines [4]. This study thus confirms the cytotoxic potency of xanthone V_1_ on a large number of cancer cell lines. The effects on xanthone V_1_ and 2-acetylfuro-1,4-naphthoquinone on the cell cycle distribution, apoptosis induction (Figs. 4 and 5) and caspase 3/7 activity (Fig. 6) were investigated in CCRF-CEM cell line. Fig. 4 shows that xanthone V_1_ was able to induce cell cycle arrest at higher concentration (2×IC_50_). At 2×IC_50_, the cell number in S-phase gradually increased (Fig. 4B1–3) with time upon treatment with xanthone V_1_, and the highest amount was observed after 72 h, suggesting a cycle arrest at this phase. Up to 8.51% and 10.53% apoptotic cells were observed after 72 h upon treatemnt of CCRF-CEM cells with xanthone V_1_ at IC_50_ and 2×IC_50_, respectively. This result is in accordance with the activation of caspases 3/7 (182.58% activation compare to untreated cells) (Fig. 6), though the effect at 2×IC_50_ was less than that obtained at a concentration corresponding to IC_50_ value (1231% activation). This is obviously due to the fact that, despite the activation of caspase induced by this compound, the ratio of cell number-activity might still be better at IC_50_ than at 2×IC_50_. Fig. 5 also shows that 2-acetylfuro-1,4-naphthoquinone induced cell cycle arrest in S-phase when tested at 2×IC_50_ and IC_50_ values in a time-dependant manner. This compound induced apoptosis, eventhough without caspase 3/7 activation (data not shown). This suggests that the activation of caspase might not be the main pathway for apoptosis induction by 2-acetylfuro-1,4-naphthoquinone.

**Table 1:** Cytotoxicity of compounds **1**, **2** and doxorubicin on different cancer cell lines.

Cell lines	Samples and IC_50_ values					
	1		2		Doxorubicin	
	µg/ml	µM	µg/ml	µM	µg/ml	µM
CCRF-CEM	4.90±0.71	12.44±1.80	9.41±2.79	35.88±11.53	**1.24±0.003**	1.28±0.005
CEM/ADR5000	19.06±2.21	48.07±5.61	16.75±0.42	69.21±1.74	>20	>36.8
PF-382	>20	>50.76	**0.57±0.06**	2.36±0.25	1.90±0.037	3.50±0.007
HL-60	4.56±0.57	11.57±1.42	4.81±0.77	19.88±3.18	1.57±0.11	2.89±0.20
MiaPaCa-2	18.56±1.76	47.11±4.47	1.81±0.02	7.48±0.08	**0.95±0.06**	1.75±0.11
Capan-1	>20	>50.76	3.81±0.96	15.74±3.97	4.06±1.18	7.47±2.17
MCF-7	**0.56±0.06**	1.42±0.15	1.66±0.09	6.86±0.72	**0.59±0.05**	1.08±0.09
SW-680	8.40±0.45	21.32±1.14	3.55±0.02	14.67±0.08	**0.93±0.09**	1.71±0.17
786-0	3.79±0.16	9.62±0.41	6.81±0.61	28.14±2.52	**0.60±0.09**	1.10±0.17
U87MG	3.80±0.01	9.64±0.03	1.94±0.18	8.02±0.74	**0.37±0.08**	0.68±0.14
A549	3.99±0.09	10.13±0.23	5.49±0.35	22.69±1.44	1.69±0.05	3.11±0.09
Colo-38	**1.19±0.07**	3.02±1.78	**0.67±0.18**	2.77±0.74	**0.81±0.06**	1.49±0.11
HeLa	**0.23±0.01**	0.58±0.03	**0.40±0.10**	1.65±0.41	**0.26±0.02**	0.48±0.04
Caski	**0.24±0.01**	0.61±0.03	**0.17±0.03**	0.70±0.12	**0.58±0.02**	1.07±0.04
AML12 (EC_50_)*	>20	>50.76	>20	>82.64	>20	>36.8

*EC_50_: effective dose showing 50% inhibition of growth proliferation.

The overall results of the present work highlight the anticancer potency of xanthone V_1_ and 2-acetylfuro-1,4-naphthoquinone, and clearly justify the fact that all compounds with any pharmacological activity should also be evaluated for its cytotoxicity. The most sensitive cancer cell lines to xanthone V_1_ and 2-acetylfuro-1,4-naphthoquinone were Colo-38 (melanoma), HeLa and Caski (cervix cancer) with IC_50_ values being closer or lower those obtained with doxorubicin. In addition, MCF-7 (breast cancer) and PF-382 (leukemia) also showed high sensitivity to xanthone V_1_ and 2-acetylfuro-1,4-naphthoquinone respectively. The cytotoxicity of acetylfuro-1,4-naphthoquinone is being reported for the first time. Regarding the medical impact of these cancers, the activities of these two molecules could be considered as very important. In fact, breast cancer is the most frequently diagnosed cancer and the leading cause of cancer death among females worldwide, accounting for 23% of the total cancer cases and 14% of the cancer deaths, meanwhile cervix and colon cancers are also amongst the most common cancer in economically developing and developped world respectively [20].

In conclusion, the results of the present study provide evidence of the cytotoxicity of xanthone V_1_ and 2-acetylfuro-1,4-naphthoquinone, and bring supportive data for future investigations that will lead to their use in cancer therapy.

## Materials and Methods

### Ethics Statement

Cell lines were obtained from different sources; Prof. Axel Sauerbrey, University of Jena, Jena, Germany: CCRF-CEM, CEM/ADR5000, HL-60; German Collection for Microorganisms and Cell Culture (DSMZ), Braunschweig, Germany: PF-382; Dr. Jörg Hoheisel, DKFZ, Heidelberg, Germany: MiaPaCa-2, Capan-1 pancreatic adenocarninoma, MCF-7 breast adenocarcinoma, SW-680 colon carcinoma cells; Tumor Bank, German Cancer Research Center (DKFZ), Heidelberg, Germany: 786-0 renal carcinoma cells, U87MG glioblastoma-astrocytoma cells, A549 lung adenocarcinoma, Caski and HeLa cervical carcinoma cells, Colo-38 skin melanoma cells; ATCC, USA: AML12 hepatocytes.

### Chemical for cytotoxicity assay

Doxorubicin (Sigma-Aldrich, Schnelldorf, Germany) was used as a positive (cytotoxic) control. The six natural compounds tested in this study are available in our research group and their isolation as well as characterization were previously reported: xanthone V_1_ C_23_H_22_O_6_ (**1**; m/z 394; m.p. 214–215, yellow powder) and physcion C_16_H_12_O_5_ (**3**; m/z 394; m.p. 202–205, orange solid) from the seeds of *Vismia laurentii* [6]; 2-acetylfuro-1,4-naphthoquinone C_14_H_8_O4 (**2**; m/z: 242; m.p. 224–225; yellow powder) from the roots bark of *Newbouldia laevis* [21]; bisvismiaquinone C_12_H_42_O_8_ (**4**; m/z: 674; m.p. 207–209; yelow powder), vismiaquinone C_21_H_20_O_5_ (**5**; m/z: 352; m.p. 201–203; red powder), 1,8-dihydroxy-3-geranyloxy-6-methylanthraquinone C_25_H_26_O_5_ (**6**; m/z: 406; m.p. 119–121; orange needle) from the twigs of *Vismia laurentii* [13]. The determination of each chemical structure was made as previously reported [6], [21], [13]. Generally they were determined on the basis of spectral data produced by one- and two-dimensional nuclear magnetic resonance (NMR), recorded on Brüker DRX-400 instrument. This spectrometer was equipped with 5 mm, ^1^H and ^13^C NMR probes operating at 400 and 100 MHz, with tetramethylsilane as internal standard. Mass spectra were recorded on an API QSTAR pulsar mass spectrometer. All chemicals were stored at 4°C before use.

### Cell lines and treatment

A panel of fourteen cancer cell lines including human CCRF-CEM leukemia cells and their multidrug-resistant subline, CEM/ADR5000, PF-382 leukemia T-cells, and HL-60 promyelocytic leukemia (moderately differentiated), MiaPaCa-2 and Capan-1pancreatic adenocarninoma, MCF-7 breast adenocarcinoma, SW-680 colon carcinoma cells, 786-0 renal carcinoma cells, U87MG glioblastoma-astrocytoma cells, A549 lung adenocarcinoma, Caski and HeLa cervical carcinoma cells, Colo-38 skin melanoma cells, as well as AML12 hepatocytes (poorly differentiated), were used. CCRF-CEM, CEM/ADR5000 and MiaPaCa-2 cells were used for the preliminary assay and the most actives compounds were tested on other cell lines. Leukemia cells were maintained in RPMI 1640 containing 100 units/ml penicillin and 100 µg/ml streptomycin and supplemented with heat-inactivated 10% fetal bovine serum (FBS). All cultured cells were maintained in a humidified environment at 37°C with 5% CO_2_. Doxorubicin (Sigma-Aldrich, Schnelldorf, Germany) was used as a positive (cytotoxic) control. The concentration of DMSO was not greater than 0.1% in all experiments.

### Resazurin cell growth inhibition assay

Alamar Blue or Resazurin (Promega, Mannheim, Germany) reduction assay [22] was used to assess the cytotoxicity of the studied samples. The assay tests cellular viability and mitochondrial function. Briefly, adherent cells were grown in tissue culture flasks, and then harvested by treating the flasks with 0.025% trypsin and 0.25 mM EDTA for 5 min. Once detached, cells were washed, counted and an aliquot (5×10^3^ ells) was placed in each well of a 96-well cell culture plate in a total volume of 100 µl. Cells were allowed to attach overnight and then treated with samples. The final concentration of samples ranged from 20-0.16 µg/ml. After 48 h, 20 µl resazurin 0.01% w/v solution was added to each well and the plates were incubated at 37°C for 1–2 h. Fluorescence was measured on an automated 96-well Infinite M2000 Pro™ plate reader (Tecan, Crailsheim, Germany) using an excitation wavelength of 544 nm and an emission wavelength of 590 nm. For leukemia cells, aliquot of 5×10^4^ cells/ml (obtained from overnight suspension) were seeded in 96-well plates, and extracts were added immediately. After 24 h incubation, plates were treated with resazurin solution as above mentioned. Doxorubicin was used as positive control. Each assay was done at least three times, with two replicates each. The viability was compared based on a comparison with untreated cells. IC_50_ (on cancer cells) or EC_50_ (on AML12 cells) values were the concentration of sample required to inhibit 50% of the cell proliferation and were calculated from a calibration curve by a linear regression [23] using Microsoft Excel.

### Flow cytometry for cell cycle analysis and detection of apoptotic cells

Leukemia CCRF-CEM cells treated with compounds **1** and **2** or DMSO (solvent control) for 24 to 72 h were fixed with ethanol 95% and washed with cold, phosphate-buffered saline (PBS; Invitrogen) and then resuspended in 150 µl hypotonic fluorochrome solution (50 µg/ml propidium iodide, 0.1% (w/v) sodium citrate and 0.1% (v/v) Triton X-100). The cells were incubated in the dark at 4°C overnight before flow-cytometry analysis was performed. The propidium iodide fluorescence of individual nuclei was measured using a FACS-Calibur cytometer (BD Biosciences, Heidelberg, Germany). Data were analyzed with the CellQuess Pro V5.2.1 software (BD Biosciences). For each condition, at least three independent experiments were performed.

### Caspase-Glo 3/7 assay

The influence of compounds **1** and **2** on caspase 3/7 activity in CCRF-CEM leukemia cell line was detected using Caspase-Glo 3/7 Assay kit (Promega). Cells cultured in RPMI were seeded in 96-well plates and treated with the sample (2×IC_50_; IC_50_; ½ IC_50_) or DMSO (solvent control). After 24 h treatment, 100 µl of caspase 3/7 reagent were added to each well, mixed and incubated for 1 h at room temperature. Luminescence was measured using well Infinite M2000 Pro™ instrument (Tecan). Caspase 3/7 activity was expressed as percentage of the untreated control.

### Angiogenesis test

#### Cultivation of quail eggs

The quail eggs were purchased from Wachtelzucht Anne Klein, Steinhagen, Germany.

The embryos were cultured according to the method described by Wittmann et al. [24]. Briefly, fertilized quail eggs were incubated for 70 h at 38°C and 80% relative humidity. After 70 h of incubation the eggs were opened. For this purpose, the eggs were placed in a vertical position to guarantee that the embryo floats in the upper part of the egg. Afterwards, hole was cut in to the top of the egg and the complete content of the egg was transferred into a Petri dish. By using this method, it could be guaranteed that the albumin gets first into the Petri dish followed by the yolk with the embryo on top without exposing the embryo to shock-forces which could damage the vitelline membrane.

### Chicken-Chorioallantoic-membran-Assay (CAM-Assay)

Compounds **1** and **2** were tested for their anti-angiogenic effects using the method of D'Arcy and Howard [25], with modifications according to Marchesan et al. [26]. Briefly, the explanted embryo was placed in an incubator for 2 h at 38°C to acclimatize to the new ambience. Subsequently, the test substance was placed on the chorioallantoic membrane (CAM). Therefore, 2% agarose solution was prepared and mixed 1∶10 with compounds prior diluted in DMSO 0.1% final concentration. The final concentration of the substance was 20 µg/ml. Pellets with 0.1% DMSO served as control. The agarose-pellets were then placed on the chorioallantoic membrane after they cooled down to room temperature. The Petri dishes with the quail embryos were placed in the incubator again and incubated at 38°C and 80% relative humidity for 24 h before documenting the effect of the applied substance.

Imaging of the vascularized quail eggs was performed using a digital camera with 3×-magnification objective (Canon eos 500 with a canon mp-e 65 2.8 macro objective). For illumination, a mercury-arc-lamp was used which provided a high fraction of blue and UV-light to obtain good contrast values between yolk and vessels. The pictured image section had a size of 5×5 mm. Following image acquisition, quantitative analysis was performed using a software routine which was written in the Image J-macro language, and the total small vessels number (or area) was then determined by the system. The percentage inhibition of vascularization was calculated as previously described [27].

### Statistical analysis

Statistical analysis of all data was performed using a Student's *t*-test or Kruskal–Wallis test followed by Dunn's post-hoc multiple comparison test (Graph-Pad Prism 5.01; GraphPad Software, Inc., CA, USA). *P*<0.05 denoted significance in all cases.
